# The Cost of Arbovirus Disease Prevention in Europe: Area-Wide Integrated Control of Tiger Mosquito, *Aedes albopictus*, in Emilia-Romagna, Northern Italy

**DOI:** 10.3390/ijerph14040444

**Published:** 2017-04-20

**Authors:** Massimo Canali, Stefano Rivas-Morales, Philippe Beutels, Claudio Venturelli

**Affiliations:** 1 Department of Agricultural and Food Science, University of Bologna, 40127 Bologna, Italy; stefano.rivasmorale2@unibo.it; 2 Centre for Health Economics Research and Modelling Infectious Diseases, Vaccine and Infectious Disease Institute, Faculty of Medicine and Health Sciences, University of Antwerp, 2610 Antwerp, Belgium; philippe.beutels@uantwerpen.be; 3 Department of Public Health, Azienda Unità Sanitaria Locale della Romagna-Cesena, 47521 Cesena, Italy; claudio.venturelli@auslromagna.it

**Keywords:** cost of area-wide integrated control of arbovirus vectors, *Aedes albopictus*, tiger mosquito, chikungunya, arbovirus prevention in Europe

## Abstract

*Aedes albopictus* (tiger mosquito) has become the most invasive mosquito species worldwide, in addition to being a well-known vector of diseases, with a proven capacity for the transmission of chikungunya and dengue viruses in Europe as well as the Zika virus in Africa and in laboratory settings. This research quantifies the cost that needs to be provided by public-health systems for area-wide prevention of arboviruses in Europe. This cost has been calculated by evaluating the expenditure of the plan for *Aedes albopictus* control set up in the Emilia-Romagna region (Northern Italy) after a chikungunya outbreak occurred in 2007. This plan involves more than 280 municipalities with a total of 4.2 million inhabitants. Public expenditure for plan implementation in 2008–2011 was examined through simple descriptive statistics. Annual expenditure was calculated to be approximately €1.3 per inhabitant, with a declining trend (from a total of €7.6 million to €5.3 million) and a significant variability at the municipality level. The preventative measures in the plan included antilarval treatments (about 75% of total expenditure), education for citizens and in schools, entomological surveillance, and emergency actions for suspected viremias. Ecological factors and the relevance of tourism showed a correlation with the territorial variability in expenditure. The median cost of one antilarval treatment in public areas was approximately €0.12 per inhabitant. Organizational aspects were also analyzed to identify possible improvements in resource use.

## 1. Introduction

*Aedes albopictus (Ae. albopictus)* is commonly known as the Asian tiger mosquito, and originates from South-East Asia. Over the last three decades, this insect has become increasingly widespread worldwide and is now considered the most invasive mosquito species [1], ranking in the top 100 invasive species of any kind [2]. It can currently be found in the temperate and tropical areas of Asia, most of the islands in the Pacific Ocean, South and Central Africa, South and North America, in addition to being found in all of Southern Europe [3,4,5,6]. The increasing international movement of people and goods has been the determinant for the global expansion of *Ae. albopictus* [5,7], which could be considered a negative consequence of international trade brought to previously unknown dimensions by globalization [8,9]. Beyond the demonstrated capacity of this mosquito to adapt to different environmental conditions, climate change has favored the current trend and is deemed to further enlarge the areas potentially exposed to the invasion [10,11,12,13,14].

The introduction of *Ae. albopictus* in Europe was probably caused by the long-distance trade of used tires, which hold rainwater and thus become perfect mosquito breeding sites [15,16]. The insect was firstly detected in Albania in 1979, followed by being found in Italy in 1990 [17,18], and is now widespread in the continent, with areas of establishment in all the countries bordering the Mediterranean basin. There are many spots of new detections in Western and Central Europe, with broader expansion expected in the future [10,11,14,19].

The popular name tiger mosquito is particularly suited for describing the appearance of *Ae. albopictus*, with its visible black and white streaks and aggressive daytime biting behavior making even a walk in a park a strenuous experience [20]. The Culicidae also attacks a large variety of mammals and birds (including domestic species), and this implies a high potential as a vector of transmissible diseases [21]. *Ae. albopictus* was identified as a main vector of the chikungunya fever virus (CHIKV) after the outbreaks in the Indian Ocean islands (2005–2007) [22,23] and in Italy (2007) [4,5,24]. It is also a well-known secondary vector for the dengue virus (DENV) [25], with equal or higher transmission competency than the primary vector *Ae. aegypti*, despite being more constrained by ecology [26]. Furthermore, *Ae. albopictus* is able to transmit the Zika virus in Africa and in laboratory settings [27,28].

Sequence analyses of the CHIKV genome revealed that the replicative capacity of the virus in the tissues of *Ae. albopictus* has been improved by relatively recent mutations. This probably facilitated the increased global frequency of chikungunya outbreaks during the last few decades, including the epidemics that occurred in Kenya (2004), Comoros (2005), and La Réunion (2005–2006) [29,30], where approximately one third of the population suffered symptoms of the disease [31,32]. The involvement of *Ae. albopictus* in localized transmission of CHIKV in Italy and France [33,34], in addition to the transmission of dengue virus in France [35] and Croatia [36], raised fears about future possible epidemics accompanying the progressive expansion of tiger mosquitoes in temperate and cold-temperate regions [30,37,38]. This highlights serious threats for the European public health systems [39].

The outbreak occurred in the year 2007 in Emilia-Romagna (ER)—a region of 22,453 km^2^ and 4.45 million inhabitants (2016) bordering the Adriatic Sea in North-East Italy—was the first proven CHIKV local vector-borne transmission in Europe. The first tiger mosquito detection in the region dates back to 1994. Since 2003, the insect has been massively present in all ER plain and hilly areas during the April–October period, and until November along the Adriatic coast [40]. In 2005, ad-hoc tiger mosquito control measures were already implemented to decrease this nuisance and prevent infectious diseases [41]. In 2007, the suspected index case was a man travelling from the Indian state of Kerala, which was affected by a CHIKV outbreak [42]. There were 217 confirmed cases in ER, with 142 cases being from Castiglione di Cervia and Castiglione di Ravenna. These are two neighboring villages (about 2200 inhabitants in total) in the lowlands south of Ravenna, where the epidemic originated. The towns of Cesena, Ravenna, Rimini, and Bologna were also affected. In total, 25 patients were hospitalized, and only one, an 83-year old man, died [38,42,43,44].

The “Regional Plan of the Emilia-Romagna Regional Health Authority for the fight against the Asian tiger mosquito and the prevention of chikungunya and dengue fevers” [40] (henceforth the “Regional Plan”) has been operating since 2008. It aims to first limit the presence of the insect in order to reduce nuisance and the risk of new outbreaks, in addition to establishing an early detection system that is able to reveal the presence of viremic patients for immediate activation of health protection measures [40,43,45]. The most important part of this plan is an area-wide integrated pest management (AW-IPM) program for the containment of the infestation. The different AW-IPM measures are implemented by municipalities, mainly on a voluntary basis. The local health authorities (LHAs) and the province administrations intervene in some activities according to local needs, while the coordination committees set up activities at the sub-regional and regional levels. The regional health authority (RHA) provides guidelines, coordinates the activities at a regional level through the LHAs, and co-finances the expenditure of municipalities.

This research evaluated the public costs related to the implementation of the Regional Plan between 2008 and 2011. The study collected data on the expenditure required by all the public administrations carrying out this plan, with the aim of assessing public spending in relation to some key indicators, in order to analyze differences in expenditure among municipalities and to examine correlations between the expenditure and relevant territorial variables. Despite the time gap from the period under analysis, the lasting scarcity of data and information about the costs to be supported by public health systems for area-wide integrated control of mosquitoes and other arbovirus vectors convinced the authors to publish this study.

## 2. Materials and Methods 

The AW-IPM activities supported by the Regional Plan for tiger mosquito control in the examined period are listed in Table 1, with the indications of the financial contribution provided by the RHA. Most of the measures are implemented between May and early October, when biting and reproduction of tiger mosquitoes are more intensive in the region. Although the plan is hierarchically coordinated at the regional level, the participation of municipalities is not compulsory, and each may individually decide which activities will be carried out in its own territory, as well as the modalities of implementation. The consequence is an extremely varied and fragmented situation in which one municipality may directly perform a given activity, but also—and much more frequently—will contract it to external operators, either by calls for tender or by direct award procedures. The types of contractors vary from large public utility corporations owned by mixed public–private investors (in the region, these organizations generally result from the merging and opening of former municipal utilities to private investors) to small private businesses. The commitments of contractors may cover several activities, only one, or just some specific tasks. Subcontracting is also largely practiced, especially by the large contractors.

Every year the municipalities and the other public administrations participating in the Regional Plan submit a technical and financial report of the activities implemented in their respective territories to the RHA in order to obtain the financial contribution. This reporting is the main data source of the study: an English translation of the reporting form filled in by municipalities in 2009–2011 is displayed in the Appendix A (Table A1). The reports display the total expenditure supported by municipalities for the implementation of the activities listed in Table 1, with the exception of entomological surveillance. This activity is fully financed by the RHA on the basis of the mosquito ovitrap checks provided by municipalities and recorded in a specific accounting manner.

In the reporting form, only some basic technical information is requested about the ordinary activities of antilarval disinfestations in public areas (i.e., the number of road drains treated and the number of treatments performed in the year) and in private areas (i.e., the number of private courtyards involved and the number of treatments performed). However, since the lack of such data does not entail a report rejection by the RHA, many municipalities provide just approximate information or no figures at all. The financing of information activity in primary schools ((f) in Table 1) is subject to a separate description of the actions performed, as well as the “other activities” undertaken by municipalities ((g) in Table 1), which are co-financed by the RHA after evaluation occurring case-by-case.

All the expenditure reported by municipalities should be accompanied by invoices and other documents testifying the payment of declared amounts, but no structured technical data are available from the reports about consumption of materials and work employed in the different activities. The dossiers submitted to the RHA are checked before the calculation of the reimbursements to be paid to municipalities according to the criteria indicated in Table 1. Furthermore, all the payments for municipalities are subject to the administrative and financial audits laid down by the law, which is the same for the RHA reimbursements.

This study excludes the overhead costs supported by public administrations for the implementation of the activities of the Regional Plan and the costs of all other activities against tiger mosquitoes undertaken by public or private organizations and individual citizens that do not receive the RHA financial support. The information available from municipality reporting and from the RHA was analyzed through simple descriptive summary statistics. Figures relative to the population were used to compare the expenditure for the planned activities at different territorial levels: municipalities, LHAs, and the whole ER region. Population data were taken from the online database of the ER regional Statistic Services [47]. Unavailability of reliable data covering the entire region prevented the use of indicators of expenditure related to the extension of the urban areas subject to larvicide treatments, as well as to the road drains treated in public areas and in private courtyards (most municipalities did not have a registry of the road drains set in public roads, and data from the financial reports were often imprecise or incomplete).

All the monetary values presented in the study correspond to the nominal values of the expenditures reported in the examined years, including the value-added tax (VAT), which is a cost for the public administrations involved (in the analyzed period, the VAT rate for the type of services taken into consideration was 20%). The findings of the analysis were discussed with the Entomological Working Group of the RHA and with the regional coordination committee for the implementation of the plan formed by representatives of the RHA and LHAs, as well as of the province and municipality administrations involved.

## 3. Results

### 3.1. The Implementation of the Plan over the ER Region’s Territory

Between 273 and 291 municipalities out of the 341–348 municipalities existing in ER took part in the Regional Plan for tiger mosquito control from 2008 to 2011 (see details in the Appendix A, Table A2). The involved population included between 4.06 and 4.28 million inhabitants, which is 95%–96.5% of the regional total (Table 2).

As mentioned, the participation of municipalities in the activities supported by the Regional Plan is not compulsory. The municipal administrations follow the RHA guidelines and coordination, but have significant autonomy in the organization of the activities they decide to implement. For these reasons, not all the municipalities participating in the plan operate all the activities listed in Table 1, and in the same way. Moreover, the range of activities executed by one municipality may change from one year to another, in addition to changes in the population involved (see Table 2).

### 3.2. Expenditure Supported for the Plan's Activities by Municipalities and the Regional Health Service

The expenditure supported by public administrations for the implementation of the different activities of the Regional Plan between 2008 and 2011 is reported in Table 3. The total expenditure decreased from €7.60 million to €5.28 million, with most of the reduction occurring between 2008 and 2009 (–22.6%) and a softer decline in the following years (–11% between 2009 and 2011). Indeed, after the 2007 CHIKV outbreak, many municipalities performed the activities of the plan very intensively, fearing new epidemics and consequent impacts on public health and economic activities, principally tourism. This resulted in higher plan expenses in 2008 than in the later years. The higher expenses in 2008 could also be attributed to the fact that this year was when province administrations and LHAs were also heavily involved in the direct implementation of the plan activities in some cases. For these reasons, the year 2008 and the relatively homogenous period 2009–2011 have been treated separately in Table 3 and further below.

The anti-larval treatments of road drains in public areas ((b) in Table 3) are the main AW-IPM activity of the plan, aimed at preventing the development of infestation hotspots in the areas that are not under the responsibility of individual citizens or private organizations. This is the only activity for which the expenditure increased after 2009, with this expenditure eventually reaching 62.4% of the total expenditure for the plan in 2011. The expenditure for the related quality controls introduced in 2009 ((d) in Table 3) covered about 6% of the plan’s expenditure over the 2009–2011 period, with an eventual decline in line with the general trend.

The door-to-door anti-larval treatments ((c) in Table 3) are intended to avoid the formation of infestation hotspots in private areas caused by negligence or ignorance of owners. This activity consists of systematic interventions in private courtyards by operators, who treat water drains, identify potential infestation hotspots, and provide advice to owners. All of this creates a high involvement of the population, but is also costly. The municipalities that implemented door-to-door anti-larval treatments were already a minority in 2008, and significantly diminished in the following years (see Table 2). The corresponding expenditure of approximately €1.13 million in 2008 almost halved in 2009, before being diminished by about one third between 2009 and 2011.

The expenditure for emergency actions to isolate viremic cases ((h) in Table 3) gradually declined to become close to zero in 2011, following a progressive decrease of alerts for these situations. The activity of entomological surveillance monitors ((a) in Table 3)—through a network of about 2700 ovitraps distributed over the ER territory—the presence of mosquitoes with respect to risk thresholds, indicating the possibility of disease transmission if viremic cases are introduced [48,49]. The corresponding expenditure was reduced by one third between 2009 and 2011, mostly as a result of a new protocol allowing a diminution of the ovitraps’ checking frequency from weekly to biweekly over the season of mosquito reproduction [46,50].

The expenses for information to citizens ((e) in Table 3), which mainly support free distribution of anti-larval kits, information materials, site inspections in private areas, and counselling, covered around 9–10% of the Regional Plan’s total expenditure over the 2009–2011 period, with a final reduction of about one quarter in the amount. Information activities in primary schools ((f) in Table 3) were introduced in 2009 to involve a selected number of school classes in the territory of each LHA every year. Beyond the education of pupils, the initiative also targets the responsiveness of their families. From 2009 to 2011, these activities accounted for around 3% of the plan’s total expenditure.

The other activities undertaken by municipalities ((g) in Table 3) embrace a variety of actions subject to a case-by-case evaluation by the RHA for financial contribution. In many cases, they are specific initiatives to improve citizens’ awareness or to develop some technical and organizational aspects of mosquito disinfestation, such as the identification, registration, and geo-referencing of the road drains in public areas. This information is relevant for the identification of potential infestation hotspots and for the management of the whole disinfestation activity, and was lacking in almost all the ER municipalities when the plan started. Under this item, the RHA also agreed only in 2008 to financially contribute to municipality expenditure for aerosol treatments against adult tiger mosquitoes in public areas for reducing insect nuisance and outside the specific emergency protocol for suspected transmissible viruses. For this reason, the item recorded the highest expenditure decrease between 2008 and 2009 (a reduction of €721 thousand). Over the 2009–2011 period, the diminution continued, but in line with the general trend and the other activities. Finally, this item was found to have contributed to about 6% of the Regional Plan’s total expenditure. 

### 3.3. The Coordination Strategy of the Regional Health Service, Financial Aspects

**Figure 1:**
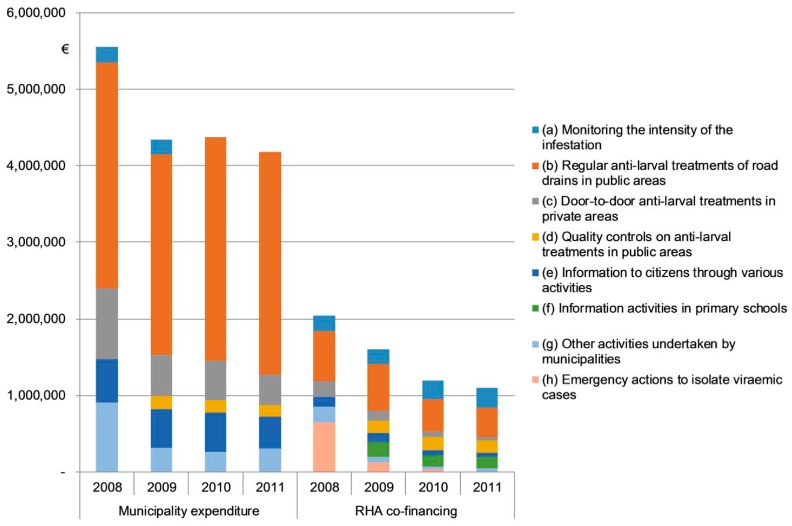
Expenditure of municipalities and the RHA for the activities of the Regional Plan for tiger mosquito control (2008–2011).

Figure 1 provides an overview of the expenditure for the Regional Plan recorded by the municipalities and the RHA, respectively. After the first year of implementation, both the municipalities and the RHA recorded an important and comparatively similar expenditure decrease (around –22%). In the following years (2010 and 2011), while the municipality expenditure was quite steady (–2.6%), the co-financing from the RHA continued to diminish significantly (–31.7%) following progressive cuts in the budget fielded for the plan by the regional government. Consequently, the municipality share in the total expenditure for the plan grew from 73.1% to 79.2%.

The differentiation of the RHA’s financial contributions among the various activities of the Regional Plan (see Table 1 and Table 3, and Figure 1) denotes a strategy aimed at stimulating municipalities to concentrate resources in anti-larval disinfestation of public areas and in information to citizens, while the provisions from the regional budget were devoted to prioritizing emergency interventions, entomological surveillance, information in primary schools, and quality controls on larvicide treatments. This strategy and the decline of emergency interventions allowed the RHA to significantly reduce the burden of the plan on its own budget, while an intensive disinfestation activity was maintained by municipalities, also in terms of financial effort. Between 2009 and 2011, the RHA financial contribution to larvicide treatments of road drains in public areas declined by 37.1%, while the expenditure of municipalities for this activity increased by 11.2% by attaining around 70% of the total municipality expenditure devoted to the plan.

### 3.4. The Territorial Variability of the Plan's Expenditure: Ecological, Economic, and Structural Factors

**Figure 2:**
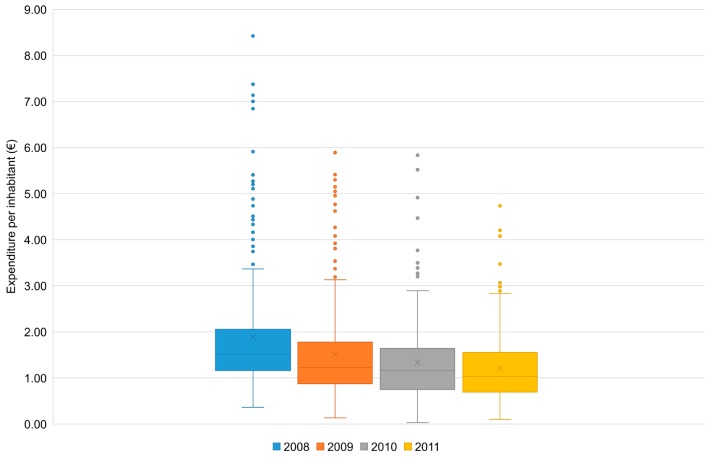
Quartile distribution of the total expenditure per inhabitant for the implementation of the tiger mosquito control Plan in the municipalities of ER (2008–2011). The evaluation included only the expenditure and the population of the municipalities that implemented the anti-larval treatments in public areas (see Table 3). With respect to the Plan’s total expenditure, they accounted for 91.5% in 2008, as well as between 97.1% and 97.6% in 2009–2011. Mean values: €1.8829 (2008); €1.5113 (2009); €1.3381 (2010); €1.2075 (2011). Coefficients of variation: 68.6% (2008); 66.6% (2009); 65.0% (2010); 63.3% (2011). Median values: €1.5137 (2008); 1.2301 (2009); 1.1633 (2010); 1.0323 (2011). IQRs: €0.8922 (2008); €0.9055 (2009); €0.8963 (2010); €0.8632 (2011).

The examined data indicate that the expenditure for the plan implementation may vary considerably from one municipality to another and from one year to another. The boxplots in Figure 2, which refer to the expenditure per inhabitant in the ER municipalities, show that there has been some decrease in the extreme values of this indicator accompanying the progressive decline of the plan spending over the analyzed years. However, the corresponding indicators of dispersion did not significantly diminish, despite starting from relatively important levels.

The spatial variability of the plan expenditure may depend on various factors. One is the changing intensity and nuisance of the tiger mosquito infestation, which is related to changes in local ecological and climatic conditions. Other factors are related to anthropic variables, such as the characteristics of the urban areas where the plan activities are implemented and the specific organization set up at the municipality level, including how many activities one municipality decides to implement and the methods these are implemented with.

The most suitable zones for mosquito infestation in the region are the coastal plain along the Adriatic Sea and the lowlands bordering the Po river, especially in the delta, which was an area of endemic malaria until the first half of the 20th Century [51]. The expenditure per inhabitant recorded for the tiger mosquito control plan was significantly higher in the territory of the LHAs that cover such areas. These are namely the LHAs of Ferrara and Ravenna, located in the delta of the Po river, as well as the LHAs of Cesena and Rimini, which include the region’s south-eastern coast, inner hills, and mountains (see Table 4).

The higher propensity of the municipalities located along the Adriatic coast to pay for the implementation of the tiger mosquito control plan may also be motivated by economic reasons. The importance of tourism in these areas seems to stimulate local administrations to strive to reduce both the level of nuisance caused by mosquitoes and the risks of disease outbreaks, which could heavily impact the presence of tourists in local seaside resorts during summertime.

Table 5 shows that the total expenditure per inhabitant for the implementation of the plan’s activities was, in most cases, significantly higher in the 12 resort municipalities located along the Adriatic coast than in respective LHAs and in the whole region. In these municipalities, a relevant implementation of anti-larval treatments in private areas contributes to the higher level of expenditure and attests to the determination of local administrations to prevent *Ae. albopictus* proliferation. The nine municipalities of the ER region characterized by important spa activities did not show similar correlations (see Table 5), despite the relevance of tourism for local economies. In fact, spa municipalities are mostly located in the foothills and in hilly or mountain areas, which are comparatively less favorable for mosquito infestations than the coastal lowlands.

### 3.5. Expenditure for Larvicide Treatments in Public Areas, the Pillar Activity of the Plan

The anti-larval treatments of road drains in public areas can be considered the pillar activity of the Regional Plan for the expected efficacy in the containment of the infestation with respect to the number of municipalities and population involved (see Table 2), and in terms of expenditure (see Table 3 and Figure 1). Thus, the territorial variability observed in the spending for the Regional Plan is strongly related to the implementation of this activity.

**Figure A1:**
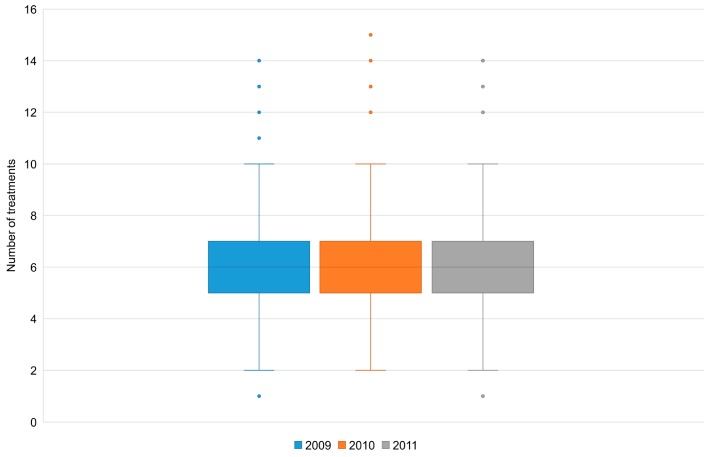
Quartile distribution of the number of anti-larval treatments in public road drains performed by ER municipalities (2009–2011). The table was elaborated with the same set of data used for Figure 3. Ranges: 1–14 treatments (2009), 2–15 treatments (2010), 1–14 treatments (2011). Median values: six treatments over the three years. IQRs: two treatments over the three years.

Road drain treatments are operated at regular intervals during the active periods of the tiger mosquito in the public spaces managed by municipalities within the urbanized areas, such as roads, squares, parks, carparks, and cemeteries. The RHA technical guidelines propose the usage of active principles and dosages allowing intervals of at least four weeks between two treatments in order to contain costs, with a minimum of four interventions in a year [40,41]. More frequent interventions may be needed due to seasonal weather trends, and the RHA experts suggest a standard of five treatments in a year as a technically correct benchmark. However, final decisions are taken at the municipality level, with the decision-making processes possibly changing significantly from one municipality to another and not necessarily following the RHA’s technical advice. This is due to other influential factors possibly intervening to induce choices for either a reduction or increase in the number of treatments, such as municipality budget constraints, advice from pest control companies, pressures from citizens, and economic operators disturbed by mosquitoes or fearing disease outbreaks, in addition to the use of active principles and dosages different from those suggested by the RHA guidelines. Consequently, in the analyzed period, there was significant variability in the number of treatments performed, and in a large majority of cases, this number was higher than the benchmark of five treatments per year indicated by the RHA experts (see Figure A1 in the Appendix A).

**Figure 3:**
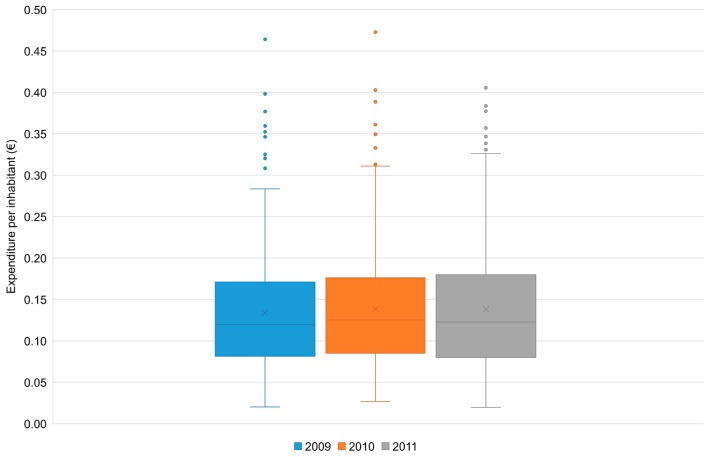
Quartile distribution of the expenditure per inhabitant supported by ER municipalities for one anti-larval treatment in public road drains (2009–2011). It was possible to elaborate the boxplots with data from 242 municipalities for the year 2009, 246 municipalities for 2010, and 249 municipalities for 2011. Respectively, they accounted for 97.7% (2009), 97.9% (2010), and 94.8% (2011) of the total population residing in the municipalities implementing this activity, and for 97.3% (2009 and 2010) and 99.1% (2011) of the total expenditure for this activity. Mean values: €0.1338 (2009); €0.1387 (2010); €0.1381 (2011). Coefficients of variation: 55.1% (2009); 54.9% (2010); 57.7% (2011). Median values: €0.1198 (2009); €0.1254 (2010); €0.1229 (2011). IQRs: €0.0896 (2009); €0.0910 (2010); €0.1000 (2011).

The calculation of the expenditure per inhabitant recorded in each municipality to operate one larvicide treatment of the road drains allowed comparisons among municipalities by isolating the variability related to the extension of the urban areas treated and to the number of treatments performed over the year. For the former, municipality population was assumed as a proxy. However, as shown in Figure 3, the level of dispersion was also important for this indicator.

The influence of the urban area size on the expenditure per inhabitant of the anti-larval treatments was tested under the hypothesis that economy-of-scale effects could be obtained by operating the service on a wider urban area and explain expenditure variability. However, the evidence of such a correlation was not found (see Table A3 in the Appendix A). In fact, the largest towns showed a tendency to attain levels of expenditure per inhabitant that were significantly higher than the medium and the small centers. This may depend on a variety of factors needing specific investigations that could not be afforded within the study.

Table 6 depicts the expenditure per inhabitant for one anti-larval treatment in the territory of the LHAs and of the whole ER region. As in the case of the total plan expenditure per inhabitant shown in Table 4, it can be observed that in the LHAs located along the Adriatic coast, the expenditure values were significantly above the regional mean, with the only exception being the LHA of Cesena. Among the seven LHAs located inland, only Reggio Emilia had expenditure values significantly above the regional mean, while the extreme low values of Parma LHA may be explained by the huge financial crisis that affected the capital municipality of this territory during the examined period.

**Figure 4:**
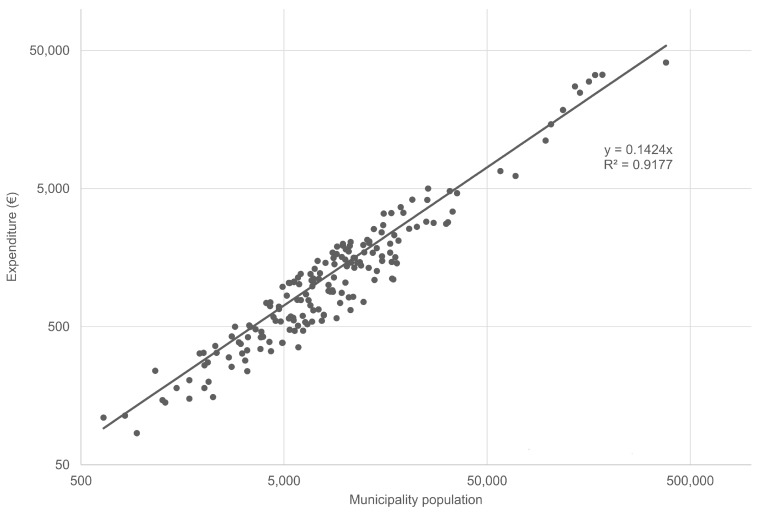
Linear regression expressing the correlation between the expenditure for one anti-larval treatment in public road drains and the demographic size of municipalities (2011). The graph was elaborated with the 2011 data of the dataset used for Figure 3, but only the values within a standard deviation from the mean value were selected. They formed a group of 176 municipalities which represented 76.3% of the population of the ER municipalities implementing anti-larval treatments in public areas and 71.1% of the total expenditure for the activity.

The differences observed in the expenditure per inhabitant of one anti-larval treatment in public road drains could also depend on the management of the plan activities at the local level, which reveal situations needing organizational improvements. The data on the expenditure per inhabitant in 2011 were used to estimate the correlation between the municipality population and the expenditure for one treatment through a linear regression. The regression equation in Figure 4 could act as an indicator for the expected expenditure for one anti-larval treatment in a municipality of the ER region with a population of *n* inhabitants in the year 2011. This allows identification of situations of possible overspending, and may stimulate actions for unravelling causes and undertaking correction measures.

For example, it was possible to compare the expenditure during 2011 for one antilarval treatment in the public areas of 249 municipalities with a benchmark set at 120% of the expected value resulting from the regression equation shown in Figure 4. The benchmark was overshot by 70 municipalities, and the overall exceeding expenditure was calculated as €334,716, which considered the total number of treatments that they actually performed in the year. This amount corresponded to the 10.2% of the total expenditure for this activity of the whole group of 249 municipalities examined.

## 4. Discussion

### 4.1. Aedes albopictus Invasion and the Role of Public Health Systems

This study analyzed the cost of an AW-IPM plan for tiger mosquito control in a region with environmental and climatic characteristics that can be found in many other areas of Europe, and where the health risks due to the presence of this vector became clear with the 2007 CHIKV outbreak. The costs to society of this bio-invasion is not limited to expenditure for pest management and health care, but should also include reduced use of recreational goods caused by mosquito nuisance, such as public and private parks and gardens. This damages users and/or owners, and may have heavy consequences on the economic activities that depend on their fruition, such as tourism [52].

If the eradication of *Ae. albopictus*—or the reduction of its population density below an epidemic risk threshold, a nuisance tolerance threshold, or a combination of both—is considered as a valuable benefit, the complex inter-causal relations that should influence public policies for mosquito control should be explored more. In the theory of public goods, the control of the *Ae. albopictus* invasion can be interpreted as a “weakest-link” problem [9,53]. This means that the effort performed by each individual actor to contain the infestation affects the result obtained by all the other actors involved, with the least effective actor (i.e., the weakest link) determining the overall level of protection for the whole community. In such a situation, individual free-rider behaviors may be particularly harmful to society. This gives strategic relevance to public health systems and enhances the role of a multi-level territorial coordination for the AW-IPM Plans. 

### 4.2. Social Cost of Ae. albopictus Invasion

An assessment of the total social cost of the *Ae. albopictus* invasion in ER would require information on many types of costs that is still unavailable: private expenses supported by households (e.g., for mosquito nets, repellents, and anti-larval treatments in private courtyards) [54], the direct and indirect damages to economic activities, the reduced utilization of parks and gardens [55,56], the health care costs for viremic cases, in addition to the related productivity and utility losses [57,58,59,60]. This study focused on the analysis of the expenditure supported by public administrations for an area-wide plan aimed at limiting tiger mosquito proliferation over a wide territory. Furthermore, some mosquito control actions performed by ER municipalities were not considered, including adulticide treatments in sensible sites such as school gardens, sport centers, and cemeteries, or in the occasion of outdoor events that concentrate large amounts of people in squares or public parks. These were not reported to the RHA in the 2009–2011 period, since they were not co-financed by the Regional Plan. Moreover, there was no information on the entity of administrative costs supported for the implementation of the plan by municipalities and other public entities involved.

### 4.3. Economic Evaluation of the Plan's Effectiveness

Despite the presence of a large-scale entomological surveillance system, there is a lack of quantitative data comparing the effects of the plan activities (e.g., reduction of mosquitoes’ density and nuisance or epidemic risk) with control areas where the same activities are not or were not performed. This lack of data prevented the possibility of evaluating the economic efficacy of the Regional Plan through cost-effectiveness or cost–benefit analysis within the framework of this study. In fact, there are no directly comparable data about the levels of tiger mosquito infestation in ER before 2008 (when the plan became operative), or about the levels of infestation in nearby regions similar for ecological and climatic characteristics where activities against tiger mosquito proliferation are not performed.

Improvements in the capacity to predict the seasonal evolution of *Ae. albopictus* population in the region with relation to changes in influencing variables (e.g., weather conditions) could allow a cost-effectiveness analysis of the plan activities by comparing the levels of infestation resulting from the ovitrap monitoring with hypothetical no-activity scenarios drawn from predictive models. However, such a capacity is not easily achievable for the well-developed area-wide monitoring system set up in the ER region, given the high influence of numerous microecological anthropic variables on density and spreading of tiger mosquitos in urban ecosystems in addition to bias caused by disinfestation activities on the possibility of using ovitrap data for those purposes [46,48]. An alternative method which is unaffordable and thus not performed in this study could be to evaluate the effects perceived by the population of the territories involved in the plan through surveys and contingent valuations. One of the very few and possibly unique cost-effectiveness and cost–benefit analysis of an AW-IPM plan for *Ae. albopictus* control followed this approach in an analysis in two counties of New Jersey. However, the dimension of the territories involved and their population (8.2 thousand inhabitants in the AW-IPM area and 13.1 thousand in the control area) were considerably smaller than the context of this study [55].

### 4.4. Recommendations for Plan Improvements

The total expenditure of the ER Regional Plan was about €7.6 million in 2008 (the year following the CHIKV outbreak), and between €5.9 million and €5.3 million in 2009–2011. This reduction was in part due to diminution of emergency interventions, which followed the decline of imported CHIKV and DENV cases. The rest of the lowered expenditure was mainly related to the reduction in the activities for citizens’ involvement, in addition to cost-saving changes in the entomological surveillance practices.

There was high variability in expenditure per inhabitant supported by municipalities for the plan implementation. This appeared to be mostly related to the large autonomy of municipality administrations for the modalities of implementation. Great differences were found in the number of activities implemented by municipalities and in the intensity of implementation of some activities. For example, the higher expenditure in coastal towns indicated the willingness to safeguard the tourism appeal for outdoor activities by reducing mosquito nuisance and the risk of infectious disease outbreaks due to their potential harm to health and to the popularity of the regional seaside resorts.

There was also high variability in municipality expenditure per inhabitant for a standard operation, such as the expenditure per inhabitant of one treatment of road drains in public areas. This could depend either on structural or casual factors that would need deeper investigations at the local level (e.g., differences either in the extension of the urban area treated or in the number of road drains relative to population). However, the cost of pesticides and other materials and tools (portable sprayers, personal protective equipment, bicycles, and other means of transport, etc.) commonly used by operators for road drains disinfestation is almost negligible with respect to the cost of the work employed and companies’ overheads. Therefore, most of the expenditure variability is probably related to organizational issues and to the capacity of municipalities to perform an effective control of prices practiced by contractors.

Some main topics about these aspects and possible improvements in the management of plan activities emerged in discussions with the experts and the representatives of municipalities participating in the regional coordination committee.

- In general, municipalities entrust the different functions related to operation, coordination, and technical control of the plan activities to external operators. It was found that one contactor undertakes all those functions in many cases. Subcontracting is also used, with obvious consequences on the ability of the municipality administrations to practice effective cost control and design cost saving strategies;
- Related to the above, it was also found that many municipalities award the services needed for the plan implementation through procedures of direct procurement. As an alternative, open tenders could offer more opportunities to reduce costs and improve efficacy;
- Municipalities often lack precise information on the road drains set in public areas. These data are necessary to optimize the management of anti-larval treatments. In the years following the period examined by this study, the RHA has been committed in helping municipalities to collect such data by ensuring partial reimbursement for identifying and geo-referencing the existing manholes and road-drains. In addition, the definitions utilized for identifying the urban areas should be standardized at the regional level, since the parameters used by municipalities were too discretional for technical and economic comparisons. A standard definition would contribute to improving the coordination of plan activities at the regional level;
- Administrative costs supported by the municipalities for the plan implementation should be taken into consideration to allow a more complete economic evaluation and a correct assessment of possible benefits coming from merging the activities of neighboring municipalities;
- Mechanisms rewarding good practices could accompany regional co-financing of municipality activities. For example, the indication of standard costs for anti-larval activities could be used to identify an acceptable range of municipality expenditure for the payment of RHA contributions. Moreover, since many municipalities seemed to operate an excessive number of treatments for road drains, the rewarding mechanism should also stimulate the fulfilment of RHA instructions and technical advice regarding the number and timing of interventions.

Further significant progress in the containment of *Ae. albopictus* infestation in ER may depend on improvements in the control activities undertaken by individual citizens in private areas, where infestation hotspots can develop with scarce possibility of rapid intervention by public authorities. Therefore, it could be useful to intensify the efforts to raise public awareness through appropriate communication strategies.

## 5. Conclusions

The ER Plan for Asian tiger mosquito control can be considered an effective initiative during the examined period. The plan’s flexibility was demonstrated by the large participation of municipalities and the amount of the population involved as well as the preservation of an intensive antilarval activity confronted with a relevant decline of the total expenditure required by public administrations. This flexibility occurred with respect to the changing conditions—environmental, but also in terms of budget, organizational capacity, and political will—of ER municipalities. When not caused by organizational inefficiencies needing correction, variability of expenditure is also an aspect of the heterogeneity of specific situations and decision-making autonomy devolved to municipalities.

With due adaptations, this plan could be proposed in other European regions already affected by *Ae. albopictus* infestations or subject to a highly probable invasion in the near future. Risk factors such as globalization and the consequent movement of people and goods worldwide will not be reduced, exacerbated by the growing concentration of population in urban areas, which facilitates the transmission of vectored viruses. Climate change expands the areas potentially suitable for the invasion of this insect, which is capable of adapting at latitudes and altitudes higher than its original habitat, and of other similar vectors even potentially more dangerous (e.g., *Ae. Aegypti*) [61,62]. On this basis, it is likely that area-wide control activities should be undertaken in other European regions, and according to the principle that integrated control has higher effectiveness in larger areas, a coordination of these initiatives at the European level may be needed.

## Figures and Tables

**Table 1:** Activities supported by the Emilia-Romagna (ER) Regional Plan for tiger mosquito control and type of financial contribution from the Regional Health Authority (2008–2011).

Activities of the Regional Plan Implemented by Municipalities	Financial Contribution of the Regional Health Authority (RHA)
(**a**) Entomological surveillance monitoring of the intensity of the tiger mosquito infestation through a network of about 2700 ovitraps distributed over the ER territory;	Lump sum paid for each ovitrap check (supposed to cover 100% of the cost supported by municipalities) *;
(**b**) Regular anti-larval treatments (from May to October) of road drains in public areas;	Variable % of the municipality expenditure, depending on the RHA budget remaining after payment of (a), (d), (f), and (h) **;
(**c**) Door-to-door anti-larval treatments in private areas;	The same as (b);
(**d**) Quality controls on the efficacy of anti-larval treatments (b) in public areas ***;	50% of the municipality expenditure;
(**e**) Information to citizens through various activities (information campaigns, free distribution of anti-larval products, inspections in private areas under request, etc.);	The same as (b);
(**f**) Information activities in primary schools ***;	Lump sum paid for each class involved (supposed to cover 100% of the cost supported by municipalities);
(**g**) Other activities undertaken by municipalities ****;	The same as (b);
(**h**) In case of the detection of potentially viremic patients, a protocol activates emergency actions to reduce the possibility of epidemic outbreaks: this includes treatments against adult mosquitoes aimed at isolating the potential outbreak hotspots;	100% of the municipality expenditure;
(**i**) Delivering of municipality ordinances requiring citizens to adopt good practices to prevent proliferation of tiger mosquitoes in private areas (courtyards, gardens, etc.);	No specific expenditure from public administrations;

* In the years 2008 and 2009, the ovitraps were checked between the end of May and early October with a frequency of once per week, and the lump sum provided by the Regional Health Authority as financial contribution (€3.5 per ovitrap check) was supposed to cover 50% of the cost of the monitoring activity. Since 2010, a technical change has allowed the monitoring to be performed by checking the ovitraps only once every two weeks [46], and the lump sum provided by the Regional Health Authority (€9 per ovitrap check) is supposed to cover 100% of the cost; ** The RHA contribution for the activities (b), (c), (e), and (g) was 18.24% of the municipality expenditure in 2008, 18.88% in 2009, 12.28% in 2010, and 11.64% in 2011; *** Activity not included in the Regional plan in the year 2008; **** Only for the year 2008, this item included the expenditure for census and cleaning of road drains and adulticide treatments in sensible sites (public parks, school gardens, cemeteries, etc.). In 2009–2011, the item includes other various actions undertaken by municipalities and admitted to the RHA financial support on a case-by-case basis.

**Table 2:** Number of municipalities implementing the different activities of the ER Regional Plan for tiger mosquito control and respective populations (2008–2011).

Activities of the Regional Plan Receiving Financial Contribution from the ER Regional Health Authority	Number of Municipalities Implementing the Activity *				% of the Total Population Involved in the Plan			
	2008	2009	2010	2011	2008	2009	2010	2011
(**a**) Entomological surveillance of the tiger mosquito infestation;	239	245	256	256	88.5	93.3	92.8	93.0
(**b**) Regular anti-larval treatments of road drains in public areas;	256	259	265	259	97.8	97.2	96.7	97.2
(**c**) Door-to-door anti-larval treatments in private areas;	76	59	52	41	25.8	30.0	25.0	18.3
(**d**) Quality controls on anti-larval treatments (b) in public areas **;	-	125	127	126	-	66.3	62.7	62.8
(**e**) Information to citizens through various activities;	190	187	179	155	77.7	72.1	82.3	69.4
(**f**) Information activities in primary schools **;	-	60	97	112	-	42.0	63.0	69.2
(**g**) Other activities undertaken by municipalities;	209	83	66	51	89.0	52.5	38.5	37.3
(**h**) Emergency actions to isolate viremic cases	83	36	14	5	41.8	26.0	26.5	13.5
**Total**	**273**	**280**	**291**	**290**	**100.0**	**100.0**	**100.0**	**100.0**
*Total population involved in the regional Plan (000 inhabitants)*	-	-	-	-	*4064*	*4178*	*4247*	*4276*

* The figures include all the municipalities receiving financial contribution from the Regional Health Authority for the implementation of the corresponding activity of the Regional Plan; ** Activity not included in the Regional plan in the year 2008; the available information on the number of municipalities and the respective population involved in this activity in the year 2009 is not complete.

**Table 3:** Expenditure for the activities of the ER Regional Plan for tiger mosquito control and share between municipalities and the Regional Health Authority (RHA) (2008–2011).

Activities of the Regional Plan	2008		2009		2010		2011		Variation 2011/2009	
	000 €	%	000 €	%	000 €	%	000 €	%	000 €	%
(**a**) Entomological surveillance of the infestation intensity *	410	5.4	386	6.5	255	4.6	258	4.9	–128	–33.3
- Municipalities **	*205*	*2.7*	*193*	*3.3*	*-*	*-*	*-*	*-*	–*193*	–*100.0*
*- RHA*	*205*	*2.7*	*193*	*3.3*	*255*	*4.6*	*258*	*4.9*	*65*	*33.5*
(**b**) Regular anti-larval treatments of road drains in public areas	3614	47.5	3226	54.3	3329	59.7	3294	62.4	68	2.1
- Municipalities **	*2955*	*38.8*	*2617*	*44.1*	*2913*	*52.3*	*2911*	*55.1*	*294*	*11.2*
*- RHA*	*659*	*8.7*	*609*	*10.3*	*415*	*7.5*	*383*	*7.3*	–*226*	–*37.1*
(**c**) Door-to-door anti-larval treatments in private areas	1129	14.8	657	11.1	590	10.6	446	8.4	–211	–32.1
- Municipalities **	*923*	*12.1*	*533*	*9.0*	*517*	*9.3*	*394*	*7.5*	–*139*	–*26.1*
*- RHA*	*206*	*2.7*	*124*	*2.1*	*74*	*1.3*	*52*	*1.0*	–*72*	–*58.2*
(**d**) Quality controls on anti-larval treatments (b) in public areas ***	-	-	340	5.7	335	6.0	308	5.8	–32	–9.4
- Municipalities **	*-*	*-*	*170*	*2.9*	*168*	*3.0*	*154*	*2.9*	–*16*	–*9.4*
*- RHA*	*-*	*-*	*170*	*2.9*	*168*	*3.0*	*154*	*2.9*	–*16*	–*9.4*
(**e**) Information to citizens through various activities	695	9.1	625	10.5	585	10.5	470	8.9	–155	–24.8
- Municipalities **	*568*	*7.5*	*507*	*8.5*	*512*	*9.2*	*415*	*7.9*	–*92*	–*18.1*
*- RHA*	*127*	*1.7*	*118*	*2.0*	*73*	*1.3*	*55*	*1.0*	–*63*	–*53.7*
(**f**) Information activities in primary schools ***	-	-	187	3.2	142	2.5	150	2.8	–37	–19.8
- Municipalities **	*-*	*-*	*-*	*-*	*-*	*-*	*-*	*-*	*-*	*-*
*- RHA*	*-*	*-*	*187*	*3.2*	*142*	*2.5*	*150*	*2.8*	–*37*	*19.8*
(**g**) Other activities undertaken by municipalities ****	1112	14.6	391	6.6	300	5.4	349	6.6	–42	–10.8
- Municipalities **	*909*	*12.0*	*317*	*5.3*	*263*	*4.7*	*308*	*5.8*	–*9*	–*2.8*
*- RHA*	*203*	*2.7*	*74*	*1.2*	*37*	*0.7*	*41*	*0.8*	–*33*	–*45.0*
(**h**) Emergency actions to isolate viremic cases	647	8.5	125	2.1	36	0.6	8	0.1	–117	–93.7
- Municipalities **	*-*	*-*	*-*	*-*	*-*	*-*	*-*	*-*	*-*	*-*
*- RHA*	*647*	*8.5*	*125*	*2.1*	*36*	*0.6*	*8*	*0.1*	–*117*	*-93.7*
(**i**) Total expenditure	7607	100.0	5937	100.0	5571	100.0	5282	100.0	–655	–11.0
- Municipalities **	*5560*	*73.1*	*4337*	*73.1*	*4371*	*78.5*	*4182*	*79.2*	–*155*	–*3.6*
*- RHA*	*2047*	*26.9*	*1600*	*26.9*	*1200*	*21.5*	*1100*	*20.8*	–*500*	–*31.2*

* For the expenditures related to this activity, it has been assumed that in the years 2010 and 2011 they corresponded to the contribution paid by the Regional Health Authority, and in the years 2008 and 2009 to twice the contribution paid (see note * in Table 1); ** In some cases, this item may include expenditure supported by province administrations and local health authorities (LHAs); *** Activity not included in the Regional plan in 2008; **** Only for the year 2008, this item includes expenditure for census and cleaning of road drains and adulticide treatments in sensible sites (public parks, school gardens, cemeteries, etc.).

**Table 4:** Total expenditure per inhabitant for the implementation of the tiger mosquito control Plan in the territory of the local health authorities (LHAs) and in the whole ER Region.

Local Health Authorities	2008 €/inhab.	2009 €/inhab.	2010 €/inhab.	2011 €/inhab.	Mean 2009–2011	
					€/inhab.	Index
Bologna	1.3774	1.3155	1.3187	1.2537	1.2960	98.0
Cesena *	2.8187	1.7832	1.5790	1.6671	1.6765	126.7
Ferrara *	2.6617	1.9805	1.6881	1.6442	1.7709	133.9
Forlì	1.1006	1.1531	1.1891	1.1078	1.1500	86.9
Imola	1.3624	1.0422	0.9827	0.8270	0.9506	71.9
Modena	1.4020	0.9708	0.8684	0.8448	0.8947	67.6
Parma	1.2004	0.8602	0.6765	0.6400	0.7256	54.8
Piacenza	1.1202	0.8371	0.7624	0.6554	0.7516	56.8
Ravenna *	2.4108	1.8225	1.7332	1.7159	1.7572	132.8
Reggio Emilia	1.5562	1.4519	1.3888	1.2034	1.3480	101.9
Rimini *	4.6222	2.7951	2.4532	2.1960	2.4814	187.6
**ER Region**	**1.8719**	**1.4210**	**1.3124**	**1.2353**	**1.3229**	**100.0**

* Territories that include coastal areas. Bold figures indicate the expenditure per inhabitant in the territory of the whole ER Region.

**Table 5:** Mean annual expenditure per inhabitant (2009–2011) for the implementation of the mosquito control plan in seaside resort and spa municipalities of the ER region.

Seaside Resort Municipalities and Respective LHA	Mean Annual Expenditure 2009–2011 (€/inhab.)		Spa Municipalities and Respective LHA	Mean Annual Expenditure 2009–2011 (€/inhab.)	
	Municipality	LHA		Municipality	LHA
Comacchio * (Ferrara)	2.3204	1.7709	Alseno (Piacenza)	0.6229	0.7516
Ravenna * (Ravenna)	1.6546	1.7572	Bobbio (Piacenza)	0.3917	0.7516
Cervia *** (Ravenna)	4.8998	1.7572	Castell‘Arquato (Piacenza)	0.3502	0.7516
Cesenatico (Cesena)	2.4851	1.6765	Montechiarugolo (Parma)	1.7070	0.7256
Gatteo *** (Cesena)	2.5512	1.6765	Salsomaggiore (Parma)	1.1918	0.7256
Savignano R. *** (Cesena)	2.7409	1.6765	Castel S.Pietro (Imola)	0.6601	0.9506
S. Mauro P. * (Cesena)	1.4487	1.6765	Brisighella (Ravenna)	0.6124	1.7572
Bellaria-I.M. * (Rimini)	1.3421	2.4814	Riolo Terme *** (Ravenna)	1.7307	1.7572
Cattolica ** (Rimini)	2.1926	2.4814	Castrocato T. (Forlì)	0.8252	1.1500
Misano A. * (Rimini)	2.3299	2.4814	Bagno di Rom. (Cesena)	0.5674	1.6765
Riccione *** (Rimini)	4.1877	2.4814	ER Region		1.3229
Rimini *** (Rimini)	2.6578	2.4814			

Asterisks indicate the number of years between 2009 and 2011 in which door-to-door anti-larval treatments were implemented in the municipality: * door-to-door anti-larval treatments implemented for one year; ** door-to-door anti-larval treatments implemented for two years; *** door-to-door anti-larval treatments implemented for three years.

**Table 6:** Expenditure per inhabitant for one anti-larval treatment in public road drains in the LHA territory of ER Region (2009–2011).

Local Health Authorities	2009 (€/inhab.)	2010 (€/inhab.)	2011 (€/inhab.)	Mean 2009–2011	
				(€/inhab.)	Index
Bologna	0.1148	0.1149	0.1136	0.1144	84.4
Cesena *	0.1027	0.1141	0.1255	0.1141	84.2
Ferrara *	0.1985	0.2327	0.2225	0.2179	160.7
Forlì	0.1389	0.1382	0.1326	0.1366	100.7
Imola	0.1126	0.1131	0.1034	0.1097	80.9
Modena	0.1103	0.1161	0.1112	0.1126	83.0
Parma	0.0752	0.0484	0.0811	0.0682	50.3
Piacenza	0.1032	0.1033	0.1060	0.1042	76.8
Ravenna *	0.1400	0.1558	0.1601	0.1520	112.1
Reggio Emilia	0.1821	0.1924	0.1745	0.1830	134.9
Rimini *	0.1478	0.1573	0.1877	0.1643	121.1
**ER Region**	**0.1305**	**0.1358**	**0.1404**	**0.1356**	**100.0**

The table was elaborated with the set of data used for Figure 3. * Territories including coastal areas. Bold figures indicate the expenditure per inhabitant in the territory of whole ER Region.

## Appendix A

**Table A1:** Form to be filled in by municipality administrations to report the annual expenditure supported for the implementation of the ER Regional Plan and to obtain co-financing by the RHA (2009–2011 period) *.

Municipality of		
Emergency actions to isolate viremic cases	(a) Total expenditure for adulticide treatments (including VAT)	€
	(b) Total expenditure for door-to-door anti-larval treatments (including VAT)	€
Treatments	(c) Number of road drains treated in public areas	
	(d) Number of anti-larval treatments performed in public areas	
	(e) Total expenditure for anti-larval treatments in public areas (including VAT)	€
	(f) Number of private courtyards involved in door-to-door anti-larval treatments (excluding treatments performed under point b)	
	(g) Number of door-to-door treatments performed (excluding treatments performed under point b)	
	(h) Total expenditure for door-to-door anti-larval treatments (including VAT and excluding treatments performed under point b)	€
Quality controls	Total expenditure for quality checks on anti-larval treatments in public areas (including VAT)	€
Information to citizens	Total expenditure for inspections performed under request in private areas (including VAT)	€
	Total expenditure for larvicide kits and information materials to be distributed to citizens (including VAT)	€
	Total expenditure for educational activities in primary schools (including VAT)	€
Other actions	Total expenditure for other actions (including VAT)	€
Total Municipality Expenditure (including VAT)		€

* Translation from the original Italian version. Invoices and other documents testifying the declared expenditure should be attached to the report.

**Table A2:** Number of municipalities participating in the ER Regional Plan for tiger mosquito control in the territory of the Local Health Authorities (2008–2011).

LHAs of ER Region	Municipalities Included in the LHA Territory	Number of Municipalities Participating in the Regional Plan *			
		2008	2009	2010	2011
Bologna	50	37	40	42	39
Cesena	15	14	14	14	15
Ferrara	26	26	26	26	26
Forlì	15	15	15	15	15
Imola	10	10	10	10	10
Modena	47	30	30	30	30
Parma	47	33	35	35	34
Piacenza	48	37	38	39	39
Ravenna	18	18	18	18	18
Reggio-Emilia	45	33	34	35	37
Rimini	20–27 **	20	20	27	27
**Total**	**341–348 ****	**273**	**280**	**291**	**290**
Population involved in the Regional Plan (inhabitants)		4,063,543	4,178,113	4,247,229	4,276,148
*- % of the ER Region’s total population:*		*95.0*	*96.3*	*96.6*	*96.5*

* The figures include all the municipalities that received financial contribution from the Regional Health Authority for the implementation of at least one activity of the Regional Plan. ** Starting from year 2010, seven new municipalities—which were formerly part of the bordering Marche Region—have been included in the territory of the LHA of Rimini.

**Table A3:** Expenditure per inhabitant for one anti-larval treatment in public road drains by population size of ER municipalities (median values 2009–2011).

Municipality Population (Inhabitants)	2009		2010		2011	
	Municipalities (n.)	Expenditure per Inhabitant, Median Values (€)	Municipalities (n.)	Expenditure per Inhabitant, Median Values (€)	Municipalities (n.)	Expenditure per Inhabitant, Median Values (€)
<3000	34	0.1430	33	0.1329	35	0.1391
3000–5000	41	0.1134	42	0.1095	40	0.1161
5000–7000	38	0.1057	40	0.1248	41	0.1099
7000–10,000	42	0.1194	43	0.1121	40	0.1065
10,000–15,000	37	0.1162	39	0.1328	39	0.1269
15,000–30,000	30	0.1277	28	0.1332	34	0.1219
30,000–90,000	10	0.1034	11	0.0900	11	0.0894
>90,000	10	0.1496	10	0.1645	9	0.1727
**Total**	**242**	**0.1198**	**246**	**0.1254**	**249**	**0.1229**

The table was elaborated with the same set of data used for Figure 3.
